# Optimization of Vagal Stimulation Protocol Based on Spontaneous Breathing Rate

**DOI:** 10.3389/fphys.2018.01341

**Published:** 2018-09-26

**Authors:** Liliane Appratto De Souza, Janaina Barcellos Ferreira, Andressa Silveira de Oliveira Schein, Daniela Ravizzoni Dartora, Adenauer Girardi Casali, Catharina M. Carvalho Scassola, Eleonora Tobaldini, Nicola Montano, Stefano Guzzetti, Alberto Porta, Maria Claudia Irigoyen, Karina Rabello Casali

**Affiliations:** ^1^Institute of Cardiology of Rio Grande do Sul, University Foundation of Cardiology, Porto Alegre, Brazil; ^2^Hypertension Division, Medicine School, Heart Institute, São Paulo University, São Paulo, Brazil; ^3^Department of Science and Technology, Institute of Science and Technology, Federal University of São Paulo, São José dos Campos, Brazil; ^4^Department of Clinical Science, Luigi Sacco Hospital, University of Milan, Milan, Italy; ^5^Department of Biomedical Sciences for Health, University of Milan, Milan, Italy; ^6^Department of Cardiothoracic, Vascular Anesthesia and Intensive Care, IRCCS Policlinico San Donato, San Donato Milanese, Milan, Italy

**Keywords:** controlled breathing, autonomic nervous system, heart rate variability, spectral analysis, vagal stimulation

## Abstract

Controlled breathing maneuver is being widely applied for cardiovascular autonomic control evaluation and cardiac vagal activation through reduction of breathing rate (BR). However, this maneuver presented contradictory results depending on the protocol and the chosen BR. These variations may be related to the individual intrinsic profile baseline sympathetic tonus, as described before by others. In this study, we evaluated the effect of controlled breathing maneuver on cardiovascular autonomic control in 26 healthy subjects allocated into two protocols: (1) controlled breathing in three different rates (10, 15, and 20 breaths/min) and (2) controlled breathing in rates normalized by the individual spontaneous breathing rate (SBR) at 100, 80, 70, and 50%. Our results showed autonomic responses favorable to vagal modulation with the lower BR maneuvers. Nevertheless, while this activation was variable using the standard protocol, all participants of the normalized protocol demonstrated an increase of vagal modulation at 80% BR (HFnu 80 = 67.5% vs. 48.2%, *p* < 0.0001). These results suggest that controlled breathing protocols to induce vagal activation should consider the SBR, being limited to values moderately lower than the baseline.

## 1. Introduction

Controlled breathing maneuver is a methodology widely used to evaluate cardiovascular autonomic control (Pinna et al., 2006; Adams et al., 2009; Stein et al., 2011; Porta et al., 2012; Ferreira et al., 2013; Krasnikov et al., 2013). Widespread evidence showing important effects of respiratory profile (rate, rhythm and amplitude) on beat-to-beat cardiovascular variability (Sakakibara and Hayano, 1996; Cooke et al., 1998; Bernardi et al., 2001; Pinna et al., 2006; Carnevali and Sgoifo, 2014; Laborde et al., 2017; Steffen et al., 2017) and the voluntary breathing control was proposed as an effective way to avoid confounding factors in short-time evaluations of cardiovascular variability (Radaelli et al., 1994; Pinna et al., 2006; DeBeck et al., 2010). Particularly, studies have verified that the breathing rate reduction is responsible for the increase of cardiac vagal modulation itself (Montano et al., 1994; Bernardi et al., 2000; Fan et al., 2011; Cabiddu et al., 2012; Tobaldini et al., 2013), which lead to the application of controlled breathing as a protocol to induce vagal activation, indicated for pathological conditions, such as hypertension (Radaelli et al., 1994; Grossman et al., 2001; Mourya et al., 2009; Gavish, 2010) and diabetes (Brown et al., 2008).

On the contrary, results associating breathing control maneuvers with lower breathing rates and the improvement of vagal modulation are still contradictory. Some studies observed an increase of cardiac sympathetic component of healthy subject in slower breathing (0.1 Hz) and an improvement of vagal component in faster breathing pattern (0.2 Hz) (Brown et al., 1993; Reimann et al., 2010). Other studies with similar protocols (controlled breathing at 0.1 and/or 0.25 Hz) did not demonstrate significant changes in blood pressure variability (BPV) and heart rate variability (HRV) (Pinna et al., 2006; Tzeng et al., 2009).

A reasonable explanation for different responses to the application of the same maneuver would be the individual variability of intrinsic autonomic profiles at the baseline. A straight relation between spontaneous breathing rate (SBR) and baseline sympathetic modulation was already demonstrated (Naughton et al., 1998; Narkiewicz et al., 2006), indicating a possible association between autonomic response to the maneuver and SBR at baseline. Meanwhile, the standardization of a normalized breathing control protocol to induce vagal activity depends on a systematic investigation of the effects of controlled breathing maneuver on cardiovascular autonomic control. This standardization would be relevant not only to the evaluation of the cardiovascular autonomic control, but also to the improvement of vagal modulation as a potential therapy for many diseases (Bernardi et al., 2001; Grossman et al., 2001; Joseph et al., 2005).

In this study we applied spectral methods to evaluate the effects of controlled breathing maneuvers on cardiovascular autonomic control in healthy subjects with different breathing rates (from 6 to 22 breaths/min), fixed or normalized to spontaneous breathe. A standard protocol, based on the normalization of individual SBR, can enhance vagal activation and this standardization could improve the efficiency and clinical applicability of vagal modulation protocols.

## 2. Materials and methods

Twenty six healthy subjects, age between 24 and 38 years old, were enrolled into the study. Recruitment and selection were done at two clinical research centers participating in this study. The participants had not been diagnosed for hypertension, diabetes, chronic respiratory disease, autonomic dysfunctions, as well as to use tobacco, alcohol or other drugs.

All participants were requested to not ingest caffeine, alcohol or practice exhaustive physical activity 12 h before the protocol. The protocol was approved, according to the ethical guidelines of the 1975 Declaration of Helsinki, by the local Committee for Ethics in Research and all subjects signed an informed consent form. After fulfill inclusion criteria, the subjects were allocated in two groups: Standard Protocol (SP; *N* = 10) or Normalized Protocol (NP; *N* = 16).

### 2.1. Standard protocol (SP)

The subjects were evaluated in the Laboratory of Clinical Investigation, Luiggi Sacco Hospital, in a silent, controlled temperature (±23°C) and illumination ambient. The electrocardiogram (ECG) was recorded at 300 Hz and the pulse pressure signal was continuously and noninvasively acquired at 1,000 Hz, in the supine position (Finapres 2300, Ohmeda, Englewood, CO). The participants of the SP were asked to breath at specific breathing rates usually found in the literature. First, the subjects should stay at rest, breathing spontaneously for 10 min while ECG would be registered. Afterwards, they should breathe following the sound generated by a digital metronome, to control breathing at different rates: 0.17 Hz (10 breathe/min, R10), 0.25 Hz (15 breathe/min, R15) e 0.33 Hz (20 breathe/min, R20). The blocks R10, R15 and R20 were set randomly. A 10-min register was performed for each protocol, with a 5 min interval between each other.

### 2.2. Normalized protocol (NP)

The subjects were evaluated in the Laboratory of Clinical Investigation, Cardiology Institute, in a silent, controlled temperature (±23°C) and illumination ambient. The pulse pressure signal was continuously and noninvasively acquired at 1,000 Hz, in the supine position (Finapres 2300, Ohmeda, Englewood, CO, United States).

In the Normalized Protocol, controlled breathing maneuvers were determined by normalization of individual SBR. First, the subjects were asked to be at rest for 10 min, while BP signals were acquired, and the SBR was observed. Afterwards, they should follow the sound generated by a digital metronome, based on individual observation, set randomly: 100, 80, 70, 60, and 50% of SBR. A 10-min register was acquired for each protocol, with a 5 min interval between each other.

### 2.3. Autonomic control assessment and evaluation

In the SP, the time series of heart rate (tachogram) were obtained from the interval between two consecutive peaks of RR interval (ECG records). In the NP, the time series of heart rate were generated by detection of the systolic peaks records of blood pressure. Stationary sequences of 200–300 beats, and coincident in both protocols, were selected (Porta et al., 2004). Frequency domain analysis of HRV and BPV was performed with an autoregressive algorithms, with emphasis on very low frequency (VLF: 0.00-0.04 Hz), low frequency (LF: 0.04–0.15 Hz) and high frequency (HF: 0.15–0.40 Hz) bands.

The spectral components were expressed in absolute (abs) and normalized units (nu). In the HRV spectrum LF and HF bands represent sympathetic and parasympathetic modulation, respectively. The ratio between LF/HF components is related to the cardiac sympathovagal balance (Malliani et al., 1991; Montano et al., 2009). Regarding the systolic blood pressure variability, the absolute LF component value represents the sympathetic vascular modulation (Stauss, 2007). The relation between the LF component of the heart rate and systolic blood pressure variability corresponds to the spontaneous baroreflex sensitivity (α_LF_ index) (Fazan et al., 2005).

Time domain indices of HRV quantify the variability between successive beats. SDNN index (ms) represents the standard deviation of normal-to-normal (NN) heart beats. The pNN50 index corresponds to percentage of adjacent NN intervals that differ from each other by more than 50 ms. The RMSSD index is calculated by the root mean square of successive differences between normal heart beats (RMSSD).

### 2.4. Statistical analysis

Data are shown as mean ± standard deviation. Statistical analyses were performed using one-way ANOVA followed by Tukey *post-hoc* test. For nonparametric data Friedman ANOVA test with repeated measures was applied. Pearson correlation was used to assess the association among variables. *P* < 0.05 was considered significant. Significant effects of the controlled breathing protocol on HRV parameters were accessed by a Linear Mixed Model (LMM). Estimation of fixed effects and covariance parameters was performed using the Restricted Maximum Likelihood (ReML) method. Null hypotheses were tested using Type III F-statistics and rejected if *p* < 0.05. Main effects of multiple-levels factors were compared using Bonferroni's adjustment of confidence intervals. Dependent variables were modeled including a fixed factor associated to the group (basal, 100, 80, 70, 60, and 50%), a random factor associated with the intercept for each subject, in order to handle the unbalanced repeated measures, and a random subject-specific effect of the respiratory maneuver. This additional random factor allows the variance of dependent variables to differ across groups. Finally, residual covariances were assumed diagonal and homogeneous.

## 3. Results

### 3.1. Standard protocol (SP)

**Figure 1:**
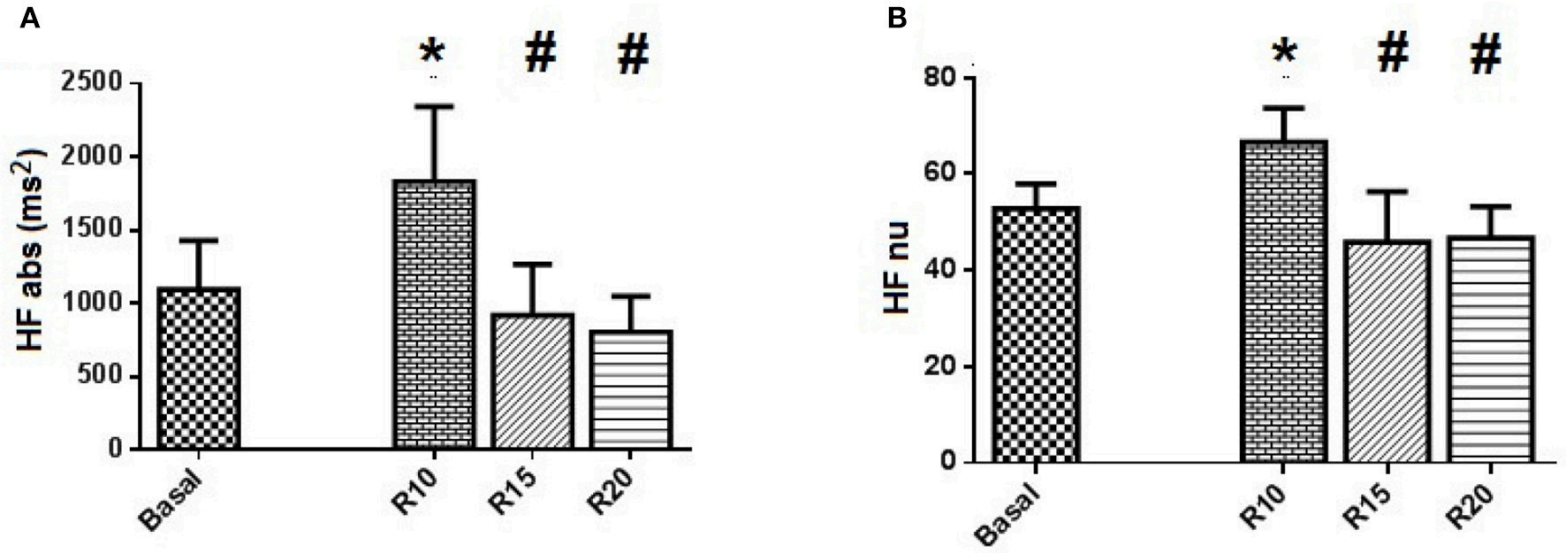
Standard Protocol Results (*N* = 10) - Spectral analysis of autonomic control applied to HRV series of SP. **(A)** shows the spectral power in absolute values of high frequency band (HFabs) and **(B)** shows the normalized values of the same component HF. Statistically significant difference compared to blocks: Basal (^*^) and R10 (#).

Ten healthy volunteers, age between 24 and 32 years old, were recruited and allocated into the SP. The subjects had a mean SBR = 14.5 ± 1.7 breathes/min. Statistical analysis between the groups of men (four) and women (six) showed no significant differences in autonomic parameters. The maneuvers did not induce changes in the values of HR and SBP (Table 1). In time domain analysis of HRV, SDNN index was reduced in R20 block. However, the assessment of autonomic control through spectral analysis showed significant changes in HRV, especially in the R10 ventilation block. Although the total spectral power value, which reports the value of HRV, was not changed, the maneuver in 10 breathe/min evoked changes related to the spectral components with increased HF band, compared to Basal, R15 and R20 blocks, both in absolute (*p* = 0.022, *p* = 0.015, and *p* = 0.011, respectively), and in normalized (*p* = 0.041) values (Figure 1). In addition, the LF/HF ratio, which is related to sympathetic-vagal balance, was lower in the R10 block in comparison to R15 and R20 (*p* = 0.028) blocks. There was no change in the parameters of VPA and its spectral components, as well as in the α index, related to spontaneous baroreflex sensitivity.

**Table 1:** Autonomic cardiovascular control of standard protocol group.

	**Basal**	**R10**	**R15**	**R20**	***p***
HR (bpm)	60.69± 10.92	60.03 ± 11.95	59.79 ± 11.17	59.43 ± 11.04	0.83
SAP (mmHg)	104.86 ± 16.04	102.57 ± 8.82	101.22 ± 13.4	104.19 ± 9.48	0.78
SDNN (mss)	57.46 ± 27.06	57.46 ± 27.03	52.09 ± 18.27	45.62 ± 15.65^*^^#^	**0.01**
RMSSD (ms)	54.82 ± 30.60	54.82 ± 30.60	52.08 ± 24.93	46.69 ± 24.30	0.31
pNN50(ms)	32.35 ± 24.64	32.35 ± 2.64	32.18 ± 24.36	24.77 ± 21.83	0.24
**Spectral analysis**					
HRV (ms^2^)	1786.18 (1057.9–4281.95)	2959.62 (1619.57–4794.65)	2797.81 (1364.5–5163.25)	1957.86 (880.13–3382.84)	0.45
LFa (ms^2^)	493.16 (246.3–1473.93)	434.51 (161.06–1004.96)	452.55 (192.82–2266.79)	518.67 (264.83–646.21)	0.23
LF nu	43.44 (33.73–51.33)	31.80 (9.87–4.21)^*^	46.42 (21.98–84.65)^#^	54.62 (34.18–63.49)^#^	**0.03**
HFa (ms^2^)	1093.92 ± 1054.26	1832.73 ± 1610.46^*^	920.37 ± 1098.35^#^	804.46 ± 773.96^#^	**0.01**
HF nu	55.71 (38.30–59.01)	66.87 (46.10–88.53)^*^	45.98 (13.62–76.12)^#^	43.81 (33.81–61.05)^#^	**0.04**
SAPV (mmHg^2^)	13.04 (5.46–21.23)	12.53 (6.1–46.67)	10.25 (4.37–24.95)	9.06 (2.45–17.31)	0.49
LFabs (mmHg^2^)	1.63 ± 1.10	5.57 ± 6.48	5.52 ± 5.69	2.32 ± 2.72	0.09
HFabs (mmHg^2^)	0.98 (0.5–1.37)	1.98 (1.4–3.81)	0.63 (0.32–0.93)	0.61 (0.46–1.05)	**0.01**
α–index	26.63 ± 20.99	14.65 ± 11.37	27.98 ± 25.36	31.20 ± 24.63	0.14

Data with normal distribution are presented as mean ± standard deviation and data with abnormal distribution are presented as median (interquartile 25% e 75%). Significant differences tested by one Way ANOVA for repeated measurements, follow by Tukey test are highlited with (^*^) compared to baseline and (^#^) compared to R10 block. Heart Rate (HR), Systolic Arterial Pressure (SAP), Standard Deviation of Normal-to-Normal (SDNN), Root Mean Square of Successive Differences (RMSSD), percentage of adjacent Normal to Normal intervals that differ from each other by more than 50 ms (pNN50), Heart Rate Variability (HRV), absolute values of RR Low and High Frequency components (LFa and HFa), normalized values of RR Low and High Frequency components (LFnu and HFnu), Systolic Arterial Pressure Variability (SAPV), absolute values of SAP Low and High Frequency components (LFabs and HFabs) and barorreflex index (α-index). Signficant (p < 0.05) p-values are displayed in bold face.

**Figure 2:**
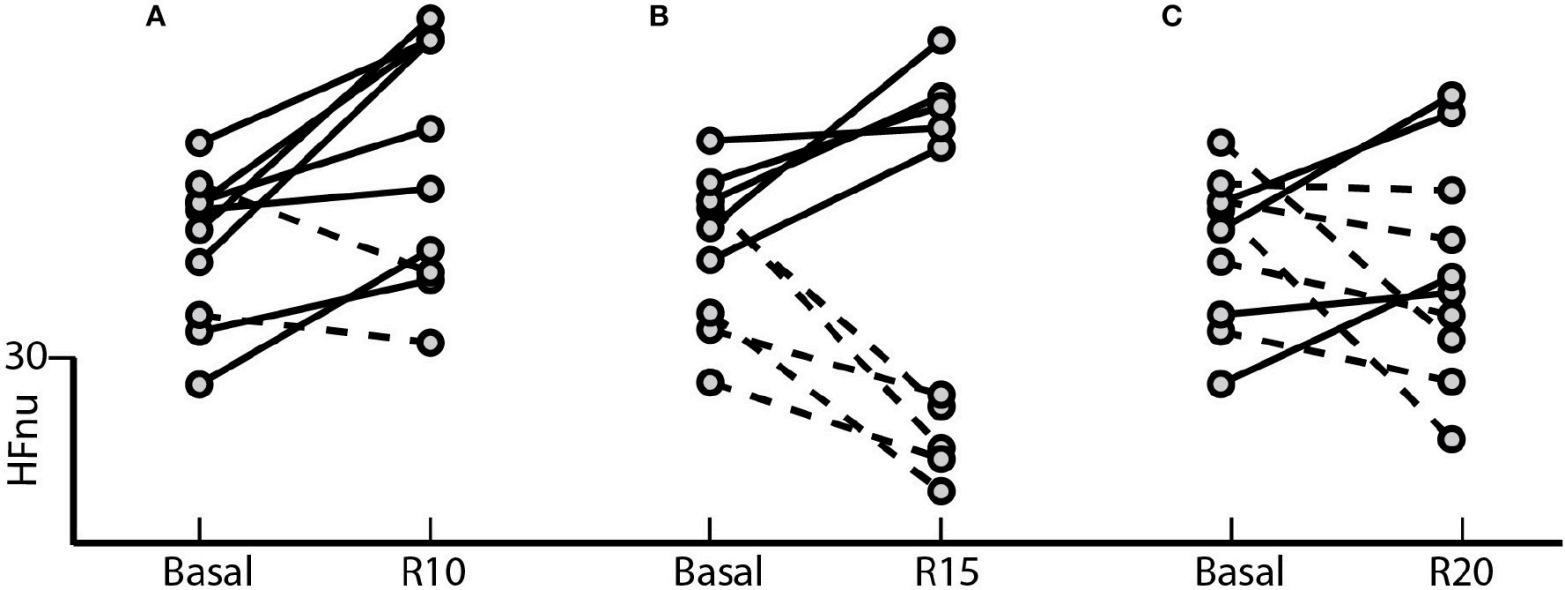
Standard Protocol Results (*N* = 10) - Effect of controlled ventilation maneuvers on the normalized HF component (HFnu) in subjects treated with SP. Solid lines denote transitions with increased HF component with respect to the baseline during the maneuver controlled ventilation. The dotted lines marked decrease in this component during the maneuver. The protocol induced an increase in HFnu on **(A)** 8 of 10 subjects in R10, **(B)** 5 of 10 in R15 and **(C)** 4 of 10 in R20.

The results indicated in Figure 2 shows the effect of controlled ventilation on the HFnu component for each subject in all different blocks of the SP. The protocol induced an increase of vagal activation in 80% of individuals R10 block (Figure 2A) in 50% R15 block (Figure 2B) and 40% of those of R20 block (Figure 2C).

### 3.2. Normalized protocol (NP)

**Figure 3:**
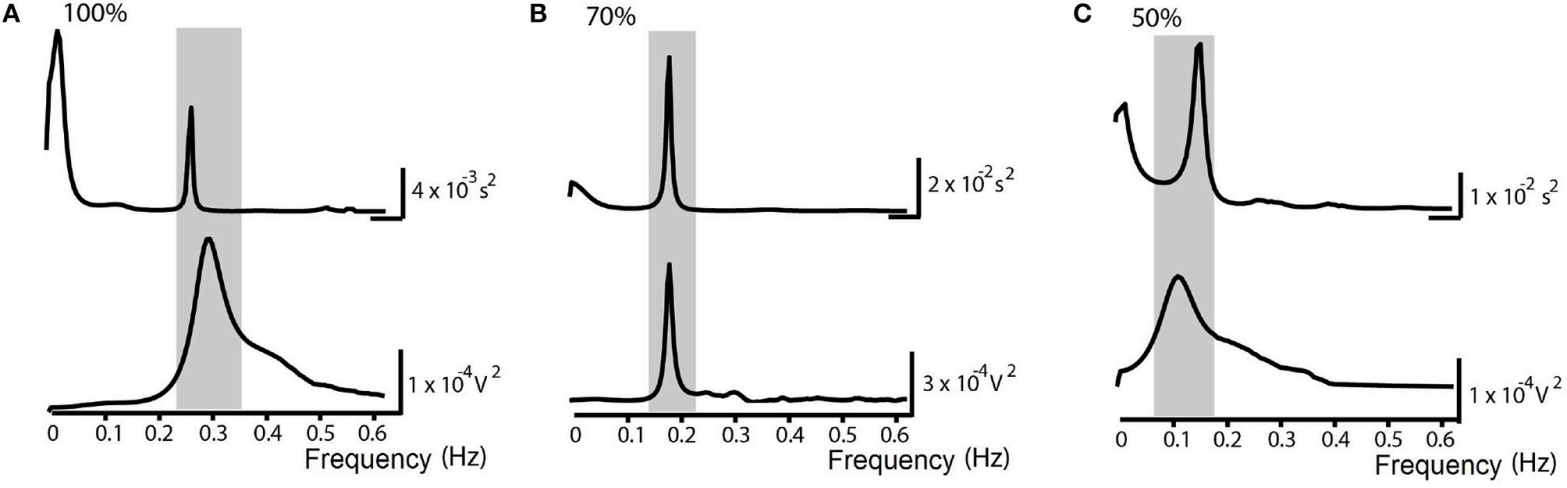
Normalized Protocol Results (*N* = 16): Spectrum examples of Heart Rate Variability (above) and the respiratory signal (below) in spontaneous breathing rate (SBR) ventilatory control situations in the normalized frequency at **(A)** 100% of the SBR, **(B)** 70% of the SBR and **(C)** 50% of the SBR. The underlined area corresponds to higher spectral coherence and demonstrates the shift of the peaks toward the called LF band (0.04-0.15 Hz).

**Figure 4:**
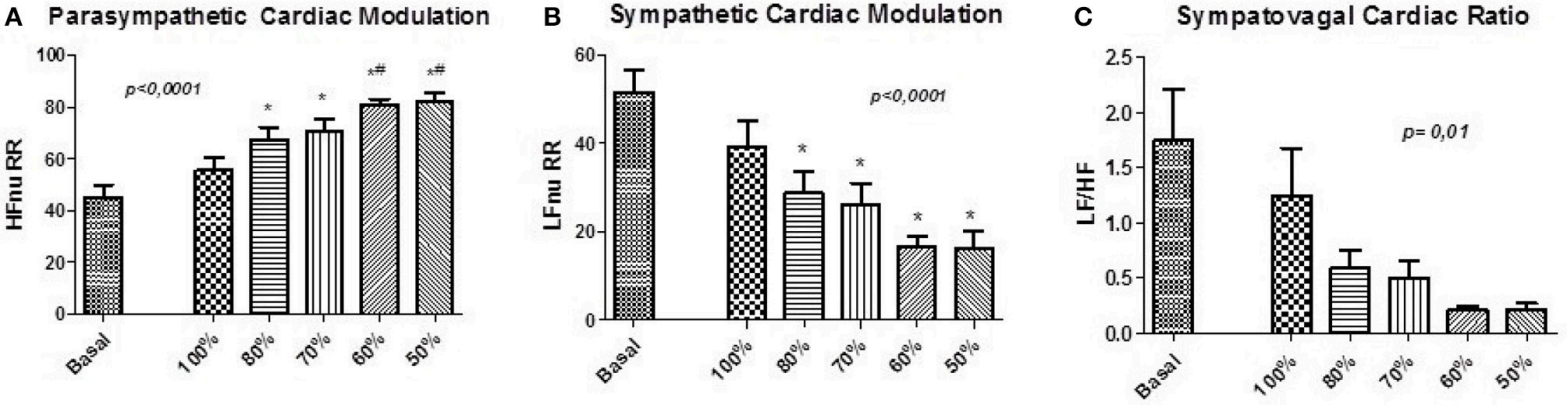
Normalized Protocol Results (*N* = 16): Parameters for the assessment of autonomic control through spectral analysis applied to the pulse interval series of NP. **(A)** shows the normalized spectral power of the high frequency HF (HFnu) band, **(B)** shows the normalized spectral power of the low frequency band LF (LFnu) and **(C)** shows the relationship between the spectral bands LF/HF. Statistically significant difference compared to blocks: Basal (^*^) and 100% (#).

**Figure 5:**
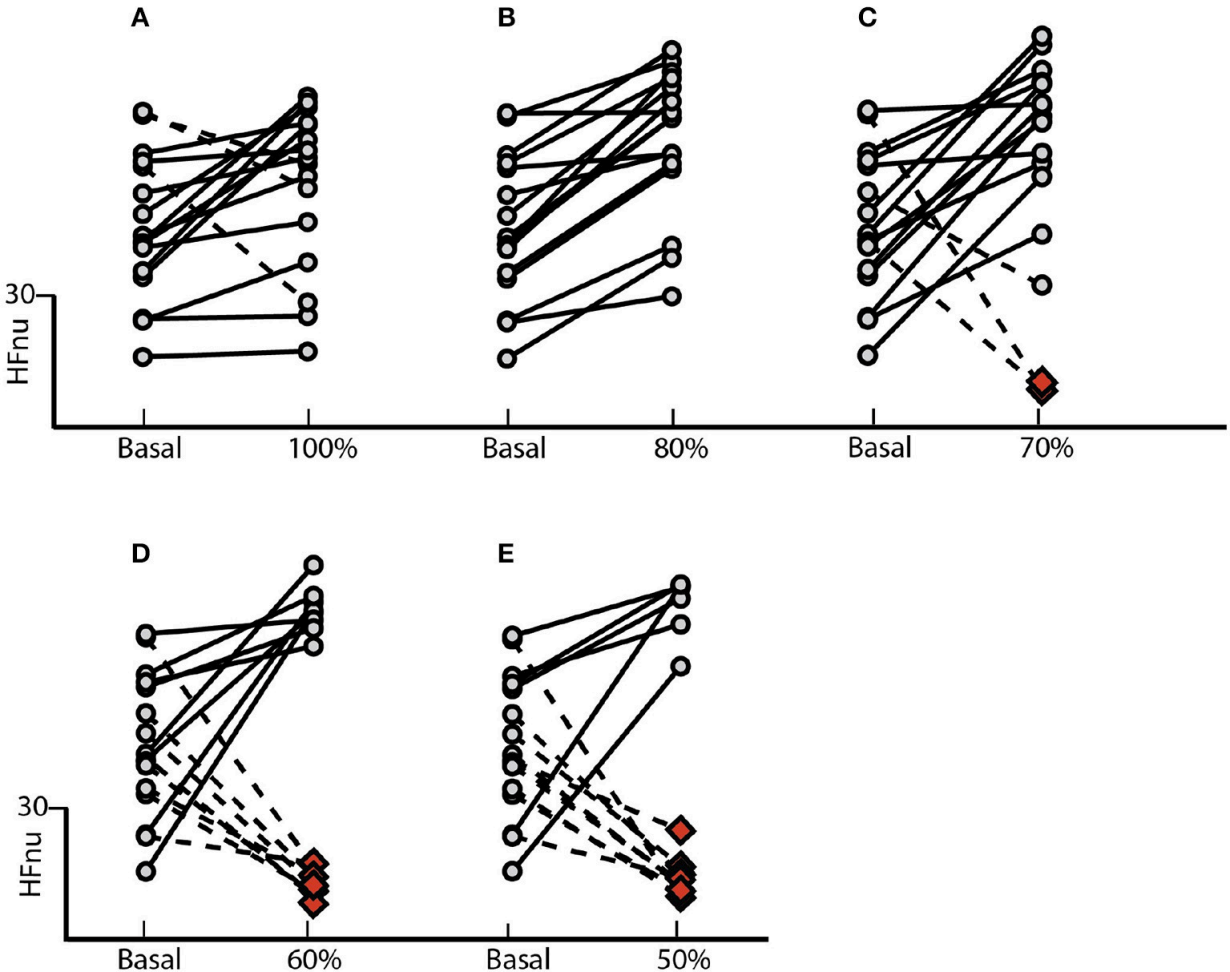
Normalized Protocol Results (*N* = 16): Effect of controlled ventilation maneuver on the normalized HF component (HFnu) in subjects treated with NP. Solid lines denote transitions with increased HF component when compared with baseline. The dotted lines marked decrease in this component. The protocol induced an increase or no change in the HF component **(A)** on 13 of 16 individuals into the group 100%, **(B)** in all 16 subjects into the group 80%, **(C)** in 13 of 16 individuals into the group 70%, **(D)** in 8 of 16 individuals into the group 60% and **(E)** in 6 of 16 individuals into the group 50%. The red dots indicate the spectral overlap of the HF band on the LF, blocking the application of such methodology.

Sixteen individuals, age between 24 and 38 years old, were recruited and allocated into the NP. The subjects studied in NP protocol presents an average SBR = 16.3 ± 2.5 breaths/min. Statistical analysis between the groups of men (six) and women (10) showed no significant differences in autonomic parameters. Blocks with imposing percentage of respiratory frequencies lower than the SBR generated displacement of HF spectral component toward the LF range. Statistical analysis between SP and NP groups at rest, showed no significant differences in autonomic parameters. Figure 3 shows the spectra of HRV and respiratory signal from one individual under situations of controlled ventilation to 100% (Figure 3A), 70% (Figure 3B), and 50% (Figure 3C) of the SBR, showing the displacement of greater coherence between the spectral band components, according to the imposed FR. Only for 100 and 80% ranges, the FR remained within the HF band in all subjects. When there is such an overlap of sympathetic and vagal frequency bands, the spectral analysis is not able to quantify both modulations apart (Malliani et al., 1991; Radaelli et al., 1994; Bernardi et al., 2000). Therefore, the results of autonomic control assessment obtained by spectral analysis are shown only for those individuals in which there was no overlap of the spectral bands while controlled ventilation maneuver was applied (Figures 4, 5 and Table 2).

**Table 2:** Autonomic Cardiovascular Control of Normalized Protocol Group.

	**Basal**	**100%**	**80%**	**70%**	**60%**	**50%**	**p**
HR (bpm)	70 ± 10	70 ± 11	69 ± 10	70 ± 10	70 ± 10	70 ± 9	0.95
SAP (mmHg)	116 ± 14	114 ± 14	118 ± 15	119 ± 14	117 ± 15	115 ± 16	0.28
SDNN (mss)	50.96 ± 21.98	53.89 ± 37.97	57.01 ± 30.91	58.63 ± 27.44	62.25 ± 31.42	60.46 ± 16.57	0.07
RMSSD (ms)	53.87 ± 39.21	50.98 ± 39.79	53.57 ± 39.18	51.21 ± 32.2	53.58 ± 35.84	55.58 ± 39.72	0.27
pNN50(ms)	23.51 ± 22.47	24.13 ± 25.05	28.45 ± 24.09	26.43 ± 22.1	26.99 ± 23.35	27.18 ± 20.32	0.34
**Spectral analysis**							
*N*	16	16	16	14	8	6	
HRV (ms^2^)	2301 ± 1931	2603 ± 2157	2877 ± 2008	3108 ± 2236	4076 ± 2914	3461 ± 1105	0.61
VLFa (ms^2^)	387.07 ± 447.81	311.87 ± 366.88	421.33 ± 471.73	465.91 ± 469.59	510.15 ± 706.83	547.1 ± 351.51	0.29
LFa (ms^2^)	875 ± 867	953 ± 1309	605 ± 483	553 ± 527	489 ± 285	427 ± 231	0.34
LF nu	48.5 ± 19.4	39.2 ± 23.3	28.6 ± 19.4^*^	26.2 ± 17.6^*^	16.8 ± 6.4^*^^#^	16.4 ± 9.2^*^^#^	<**0.0001**
HFa (ms^2^)	976 ± 998	1253 ± 1203	1759 ± 1479	1992 ± 1768	2817 ± 2109	2292 ± 1022	0.05
HF nu	48.2 ± 18.7	55.4 ± 20.8	67.5 ± 18.8^*^	70.4 ± 18.1^*^^#^	81 ± 6.4^*^^#^	82.3 ± 8.7^*^^#^	<**0.0001**
LF/HF	1.62 ± 1.85	1.25 ± 1.72	0.59 ± 0.67^*^	0.50 ± 0.58^*^	0.21 ± 0.09^*^	0.21 ± 0.15	**0.01**
SAPV (mmHg^2^)	23.2 ± 24.0	22.1 ± 17.3	26.9 ± 24.9	30.8 ± 30.5	28.3 ± 17.8	42.1 ± 37.0	0.47
LFabs (mmHg^2^)	5.6 ± 12.5	6.9 ± 9.6	15.4 ± 25.6	9.6 ± 14.9	12.9 ± 17.4	18.0 ± 21.0	0.32
α-index	12.6 ± 5.5	15.2 ± 12.0	14.5 ± 11.1	13.4 ± 11.7	14.6 ± 17.8	13.2 ± 16.7	0.69

Data are presented as mean ± standard deviation and data with abnormal distribution were analyzed through Shapiro Wilk Test. Statistic (p) from Linear Mixed Model (LMM). Significant multiple comparisons corrected by Bonferroni's method: (^*^) compared to baseline and (^#^) compared to 100% block. Heart Rate (HR), Systolic Arterial Pressure (SAP), Standard Deviation of Normal-to-Normal (SDNN), Root Mean Square of Successive Differences (RMSSD), percentage of adjacent Normal to Normal intervals that differ from each other by more than 50 ms (pNN50), Heart Rate Variability (HRV), absolute values of RR Very Low, Low and High Frequency components (VLFa, LFa and HFa), normalized values of RR Low and High Frequency components (LFnu and HFnu), relation between RR LF and HF components (LF/HF), Systolic Arterial Pressure Variability (SAPV), absolute values of SAP Low Frequency component (LFabs) and barorreflex index (α-index). Signficant (p < 0.05) p-values are displayed in bold face.

There were no changes in HR and SBP, as well as in the values of HRV and BPV components. The assessment of autonomic control demonstrated changes in the LF and HF components, related to sympathetic and cardiac vagal modulation, respectively (Table 2, Figures 4A,B, 5). Furthermore, the ventilation control lead to increase of HFnu component (*p* < 0.0001), reduction of LFnu component (*p* < 0.0001) and of LF/HF index (*p* = 0.01) of all VC sessions in relation to baseline. There were no changes in the spectral BPV components for any sessions to which subjects underwent. However, when evaluated individually it was observed that the responses did not occur homogeneously in every situation.

The effect of the NP on the HFnu component of all ventilation blocks for each subject individually is observed in Figure 5. The protocol induced an increase of vagal activation or no changes of HFnu component in 81% of subjects underwent to controlled ventilation in SBR (Figure 5A). On the 80% block ventilation of SBR, all subjects showed such results (Figure 5B). In addition, a total of 81% of subjects responded with activation of vagal modulation or no change in the component HFnu block ventilation to 70% of RES (Figure 5C) as well as half of the subjects of the ventilating block 60% of the SBR (Figure 5D) responded similarly only 37% of subjects subjected to block ventilation of 50%.

## 4. Discussion

The controlled ventilation maneuver lead to vagal activation and it is indicated in the treatment of hypertension, in order to reduce blood pressure and improve the clinical profile (Radaelli et al., 1994; Sakakibara and Hayano, 1996; Cooke et al., 1998; Grossman et al., 2001; Pinna et al., 2006; Reimann et al., 2010). Several studies, however, consider absolute respiratory frequencies of 10 or 15 breaths/min to be applied indiscriminately in many situations. The respiratory rate indicated by the Brazilian Guidelines of Hypertension, for example, is 10 breaths/min (SocBrasCardio, 2010).

Our results highlight for the very first time the importance of considering individual spontaneous respiratory rate and its effect on basal sympathetic tone and vagal activation. Although we observed a significant increase in vagal activation at 10 breaths/min (Figure 1), the individual analysis of subjects treated with the SP (Figure 2) shows the heterogeneity of the responses within this group, with increased vagal activation in 80% of subjects in R10, 50% of R15 individuals and 40% of those of R20. When we take into account the individual SBR and apply the normalized protocol, vagal modulation was already increased in 81% of subjects just by controlling the ventilation at individual SBRs (Figure 5A). Moreover, vagal activation was achieved with homogeneity in all individuals when breathing at a rate of 80% SBR (Figure 5B).

It is known that slow breathing induces a generalized decrease of excitatory pathways in the regulation of cardiovascular and respiratory systems (Joseph et al., 2005; Laborde et al., 2017; Steffen et al., 2017). Some authors have shown that controlled ventilation, particularly slow breathing 3-6 breaths/min, reduces HR and BP both in healthy individuals (Sakakibara and Hayano, 1996; Krasnikov et al., 2013) and hypertensive patients (Joseph et al., 2005). The mechanisms involved in the changes imposed by slow ventilation, however, are not yet fully known. Some authors attribute these effects to reflex changes, such as increased baroreflex sensitivity (Bernardi et al., 2001; Joseph et al., 2005; Reimann et al., 2010) and reduction in chemoreflex. Anatomical changes of pulmonary distress may also evoke autonomic responses (Bernardi et al., 2001; Cabiddu et al., 2012) and the Hering-Breuer reflex, which induces inhibition of inspiration after inflation of the lung, is also mediated by vagal innervation and appears to have an important role in the regulation of respiration and respiratory depth (Clark and von Euler, 1972). Our results showed no changes in spontaneous baroreflex sensitivity, which can be attributed to an indirect evaluation method (Fazan et al., 2005) or to methodological variations (Tzeng et al., 2009). In any event, our results suggest that the order and magnitude of these possible mechanisms of action during the operation of controlled ventilation depend on individual characteristics such as spontaneous breathing frequency. In the group to which it was imposed a fixed respiratory rate of 10 breaths/min, we could find individuals breathing at their own SBR and others breathing at a rate near 50% of their SBR. Our study supports the hypothesis that this maneuver have different effects in the two cases: in these latter individuals such a maneuver would not only be neffective in reducing BP, but could also evoke sympathetic activation and reduce vagal modulation.

Recent study evaluated the effects of gradual increase and decrease of breathing rate on healthy subjects and appointed that, in the few subjects who had significant trends, sympathetic burst areas could change directly or inversely with breathing frequency (Stankovski et al., 2013).

Protocols that impose very slow breathing rates can induce physiological changes in lung function in search of adaptation to prolonged ventilation. Such changes may occur acutely and are deleted when the protocol involves training with evaluation of chronic effects. Both hypoxia and hypercapnia increase ventilation and can increase sympathetic tone, HR and SBP (Somers et al., 1989a,b; Van de Borne et al., 2000). Our study did not evaluate the parameters of respiratory function due to the fact that the methodology be able to insert autonomic changes confounding the analysis (Bernardi et al., 2000).

A possible increase in cardiac sympathetic component has importance both as investigative clinical character once controlled ventilation has been applied as drug intervention in several diseases such as in hypertension (Radaelli et al., 1994; Grossman et al., 2001; Mourya et al., 2009; Gavish, 2010), diabetes (Brown et al., 2008) and acute myocardial infarction (Adams et al., 2009). Abnormal respiratory modulation is often related to autonomic dysfunction and manipulation of the breathing pattern can promote beneficial effects for the cardiovascular and respiratory control in both physiological and pathological conditions effects. Thus, it opens a new field for future research aiming at improving the management of patients with cardiovascular autonomic dysfunction (Bernardi et al., 2001). However, the success of the method depends largely on the interaction of the patient and the pathological conditions (Gavish, 2010).

## 5. Conclusion

In conclusion, we have shown the importance of SBR in the evaluation for the application of maneuvers controlled ventilation. We demonstrate the behavior of the autonomic control with the variation of RF, by proposing a standard baseline for each subject protocol. The use of the maneuver of controlled ventilation at 80% of SBR evoked increase in cardiac vagal component in all subjects assessed, enhancing its application in protocols designed to improve vagal activation in healthy individuals, in view of the importance of applying a method with known effect and the most reliable possible, and motivating the study of such a protocol in pathological situations.

## 6. Outlook

As perspectives, the standard protocol could be applied to populations of different pathologies for both assessments for chronic and acute effect study by adaptively training, seeking the construction of individual models for more efficient responses. Furthermore, the study of the acute effect of slow BR with methodological rigor, evaluating directly the sympathetic tone and respiratory function, could provide more precise information about the effect of FR at 50% of SBR on cardiovascular autonomic control.

## 7. Limitations

We admit as an important limitation of this study, the lack of respiratory function evaluation, such as spirometry. We understand that this evaluation could lead to more detailed analysis on the possible physiological adaptations during maneuvers. In addition, we could be more accurate in the HRV analysis if we also had ECG records.

## Author contributions

KC, MI, and NM conceived and designed this study. LD, JF, AS, DD, ET, SG, and AP collected the data and LD, and AC analyzed it. LS, AC, CS, and KC drafted the article. DD, AC, CS, NM, MI, and KC made the critical revision of the article. All authors approved the final version.

### Conflict of interest statement

The authors declare that the research was conducted in the absence of any commercial or financial relationships that could be construed as a potential conflict of interest.
